# Exploring the effects of peripheral sensibility on visuospatial and postural capacities during goal-directed movements in long-term Tai Chi practitioners

**DOI:** 10.3389/fnagi.2022.881972

**Published:** 2022-07-22

**Authors:** Zhufeng Shao, Li Li, Min Mao, Wei Sun, Cui Zhang, Qipeng Song

**Affiliations:** ^1^College of Sports and Health, Shandong Sport University, Jinan, China; ^2^Department of Health Sciences and Kinesiology, Georgia Southern University, Statesboro, GA, United States; ^3^School of Nursing and Rehabilitation, Cheeloo College of Medicine, Shandong University, Jinan, China; ^4^Laboratory of Biomechanics, Shandong Institute of Sport Science, Jinan, China

**Keywords:** coordination, postural balance, proprioception, tactile sensation, Tai Chi quan

## Abstract

**Background:**

Falls are directly related to visuospatial ability and postural stability. Perturbations of upper body movements pose a challenge to older adults and may cause falls. This study investigated visuospatial ability and postural stability during goal-directed upper body movements between the Tai Chi and control groups and tried to connect them with their sensations.

**Materials and methods:**

Thirty-seven older adults were recruited to perform the touch (TT) and blind touch (BTT) tasks. The target positioning error (TPE), ankle proprioception, tactile sensation, time to stabilization (TTS), and maximum displacement (D_max_) of the center of pressure trajectory were compared between the groups during the tasks. The relationships of visuospatial ability and postural stability to proprioception and tactile sensation were investigated.

**Results:**

D_max_ in the mediolateral (D_maxML_) direction decreased during BTT compared to TT among the Tai Chi group but not the control group. Compared to the control group, less D_max_ in the anterio-posterior (D_maxAP_) direction, and shorter TTS in AP/ML (TTS_AP_/TTS_ML_) directions were observed among the Tai Chi group. Compared to TT, D_maxAP_ decreased during the BTT. The Tai Chi group had less TPE in the vertical (TPE_V_) direction and in three-dimensional space. Among the Tai Chi group, TPE_V_, TTS_ML_, and D_maxAP_ were correlated to their proprioception during plantarflexion; TTS_AP_ was correlated to tactile sensation at the great toe during the TT and BTT; D_maxAP_ was correlated to tactile sensation at the great toe during the TT. Among the control group, TTS_ML_ was correlated to ankle proprioception during dorsiflexion and plantarflexion during the BTT.

**Conclusion:**

Long-term Tai Chi practitioners exhibited superior visuospatial ability and postural stability during goal-directed upper body movements, which was associated with sensitive proprioception and tactile sensation.

## Introduction

Falls in older adults pose a severe problem and are the fifth leading cause of death, following cancer, heart disease, stroke, and respiratory diseases (Canada, 2011). Standing with goal-directed upper body movements (reaching and fitting) is an important activity in daily life and is closely associated with motor, sensory, or cognitive functions among older adults (Hyndman et al., 2002; Pan et al., 2016). Goal-directed upper body reaching movements require the visuospatial ability of individuals (Hondzinski et al., 2010), and the perturbations of upper body movements under such tasks challenge the postural stability among older adults (Pan et al., 2016).

Falls are directly related to visuospatial ability (Naslund, 2010) and postural stability (Liu et al., 2012), especially during goal-directed upper body movements. Visuospatial ability is often used in describing how the mind organizes and understands two- and three-dimensional spaces (Pinker, 1984) and is one of the essential abilities in daily life (Martin et al., 2009). Poor visuospatial ability is associated with a greater risk of falls among older adults (Naslund, 2010). The target positioning error (TPE) measured during a blind touch task (BTT) could be used as an important indicator for evaluating visuospatial ability (McIntyre et al., 2000), indicated by a distance between the participant’s pointed position by memory and the original target position after the target was removed. Less TPE indicated better visuospatial ability (McIntyre et al., 2000). Postural stability was often used to reflect an individual’s ability to control their posture during locomotion (Kang and Dingwell, 2006). A proper postural control strategy was needed to restore the body’s stability when needed (Haddad et al., 2012). Greater postural stability enhanced the ability to resist perturbations, thereby reducing the risk of falls (Latash et al., 2010). Postural stability could be reflected by time to stabilization (TTS) (Ross and Guskiewicz, 2003, 2004; Fransz et al., 2015) and maximum displacement (D_max_) of the center of pressure (COP) trajectory during goal-directed upper body movements (Prieto et al., 1996; Pan et al., 2016). Shorter TTS (Fransz et al., 2015; Sherrington et al., 2019) or the less D_max_ indicated better postural stability and reduction in fall risk (Prieto et al., 1996; Pan et al., 2016).

A few types of physical activities are effective in reducing fall risk among older adults (Bangsbo et al., 2019). As a traditional Chinese multi-genre fitness exercise, Tai Chi might be an option for improving visuospatial ability and postural stability. Visuospatial ability (Nemoto et al., 2020) and postural stability (Ghai et al., 2017) requires combinations of cognition and movement coordination. Tai Chi encompasses motor (a series of body movements) and mental (mind concentration) elements (Wayne et al., 2014; Song et al., 2018), and has been proven to improve motor and cognitive functions among older adults (Solianik et al., 2021). Further, practicing Tai Chi improved the sensitivity of proprioception and tactile sensation (Sun et al., 2015; Hu et al., 2021), which were positively related to visuospatial ability (Hondzinski et al., 2010) and postural stability (Zhang et al., 2015). Moreover, Tai Chi involves upper body movements supported by lower extremities to maintain postural stability, similar to the goal-directed upper body movements. Therefore, visuospatial ability and postural stability during goal-directed upper body movements may benefit from Tai Chi practice.

As individual ages, their visuospatial ability and postural stability decline rapidly, twice as fast as the decline in memory (Murre et al., 2013; Lee et al., 2019). Therefore, a suitable exercise that delays or reverses a decline in visuospatial ability and postural stability among older adults is urgently needed. Although the relationship of visuospatial ability and postural stability to sensations has been investigated, the different roles of its two main components, proprioception, and tactile sensation, have yet been fully understood. Therefore, the purpose of this study was to investigate the benefits of Tai Chi practice for visuospatial ability and postural stability during goal-directed upper body movements and their relationship to practitioners’ visuospatial ability and postural stability were related to their proprioception and tactile sensations. It is hypothesis that 1. compared to the control group, Tai Chi practitioners have better visuospatial ability and postural stability; 2. The visuospatial ability and postural stability are positively correlated with the sensitivity of proprioception and tactile sensation.

## Materials and methods

The study design is analytical cross-sectional.

### Participants

An *a priori* power analysis (G*Power Version 3.1) indicated that a minimum of 15 participants was needed in each group to obtain an alpha level of 0.05 and a beta level of 0.80 based on a previous report, in which the TTS was compared after the matched bias (2.75 ± 0.85) or unmatched bias (3.76 ± 1.01) exercises (Tulloch et al., 2012). The current study recruited 37 older adults aged 65∼77 years through flyers, leaflets, and advocacy from local communities (Table 1). The inclusion criteria were as follows: age ≥ 65 years, long-term Tai Chi practice experience (at least four times per week, 1 h each time, for more than 5 years) for the Tai Chi group, and absence of regular exercise (total exercise time less than 1 h per week in the past 3 years) for the control group. The exclusion criteria were movement disorders or nervous system diseases, recent lower extremity and dominant arm surgery, cardiovascular pathologies, diabetes or hepatorenal syndrome, coordination function disorders, peripheral neuritis, Parkinson’s disease, Alzheimer’s disease, and Mini-Mental State Examination (MMSE) scores < 24. All participants were right arm dominant, defined by the outstretched hand to reach an object (Haddad et al., 2008). All the participants signed informed consent forms before the formal test. The project was approved by the Ethics Committee of Shandong Sports University (2020108) and in accordance with the Declaration of Helsinki.

**TABLE 1:** Basic information of the participants in Tai Chi and control groups.

Tai Chi group						Control group				
Sex	Height (cm)	Weight (kg)	BMI (kg/m^2^)	Age (years)	EXP (years)	Sex	Height (cm)	Weight (kg)	BMI (kg/m^2^)	Age (years)
Male	181	93.4	28.51	72.7	8.1	Male	170	68.2	23.60	75.2
Male	165	66.5	24.43	68.6	8.9	Male	162	63.6	24.23	65.0
Male	161	60.6	23.38	70.5	5.2	Female	156	71.3	29.30	71.8
Male	180	76.0	23.46	77.3	10.8	Female	156	71.4	29.34	67.7
Male	170	75.0	25.95	72.9	19.6	Male	160	62.1	24.26	66.3
Male	164	66.1	24.58	76.4	20.4	Male	168	82.9	29.37	70.2
Male	171	86.0	29.41	65.4	5.4	Female	153	44.7	19.10	67.8
Male	171	78.0	26.67	67.4	9.6	Male	177	89.9	28.70	69.6
Male	168	71.1	25.19	65.2	5.3	Male	164	64.7	24.06	76.0
Female	156	71.9	29.54	65.6	6.0	Male	164	56.3	20.93	65.4
Male	174	72.8	24.05	65.3	5.7	Female	152	64.0	27.70	67.2
Male	173	81.0	27.06	73.1	11.8	Female	154	56.3	23.74	68.7
Female	158	56.3	22.55	72.6	21.2	Male	176	90.8	29.31	66.1
Male	176	76.7	24.76	75.4	20.3	Male	166	75.1	27.25	65.7
Female	155	48.6	20.23	67.7	5.5	Male	175	84.8	27.69	65.6
Male	171	74.3	25.41	66.9	8.2	Male	166	76.3	27.69	69.7
Female	157	57.6	23.37	66.4	9.2	Male	164	71.1	26.44	65.5
Male	181	59.1	18.04	65.6	9.8	Male	168	75.2	26.64	66.4
–	–	–	–	–	–	Male	175	91.0	29.71	66.1
AVE	168.4	70.6	24.81	69.7	10.6	AVE	164.5	71.4	26.2	68.2

EXP, experience; BMI, body mass index; M, male; F, female; AVE, average.

### Testing protocol

#### Touch task and blind touch task

Each participant wore experimental shoes provided by the laboratory (Flattie, Qingdao Luzhong Co. Ltd., Qingdao, China). A reflective marker (Marker-1) was attached to a metal bar (height = 2 m, diameter = 1.5 cm) with a solid base; another reflective marker (Marker-2) was attached to the tip of the index finger of a participant’s dominant arm. The metal bar was removable, and the height of Maker-1 was adjustable. A force plate (AMTI 600*900, AMTI Inc., Watertown, MA, United States) was used to collect force data at 1,000 Hz. A 12-camera motion analysis system (Vicon, Oxford Metrics, Yarnton, England) was used to collect the markers’ three-dimensional data at 100 Hz. The force plate and motion analysis system were collected via the Vicon system with internal synchronization.

**FIGURE 1:**
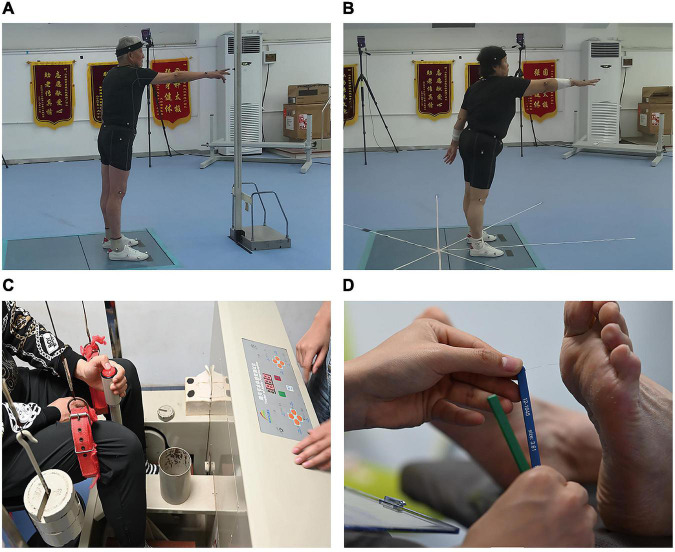
The illustration about the actual testing protocol. **(A)** Touch task; **(B)** blind touch task; **(C)** proprioception test; **(D)** tactile sensation tests.

The location of the marker-1 is adjustable. The horizontal location could be adjusted by moving the bar forward or backward, the vertical location could be adjusted by attaching the marker to the high or low part of the bar. The height of Marker-1 was adjusted to the participant’s shoulder joint height with 1.3 times the dominant arm length to the shoulder joint horizontally. The metal bar location was adjusted to allow participants to stand on the center of the force plate. Each participant was asked to complete 2 tasks in a fixed order, namely, touch task (TT) and BTT. During the TT (Figure 1A), each participant stood quietly on the force plate for 30 s for stabilization. When the command “start” was given, the participants raised their dominant arms and used Marker-2 to touch Marker-1 at a comfortable speed, then brought their arms back to the initial position and stood still for another 30 s. During the BTT (Figure 1B), the metal bar with Marker-1 was removed from sight manually about 1 s before individual movement, and the participants could see the process of moving out (Hondzinski et al., 2010). The participants raised their dominant arms and used Marker-2 to point to the remembered position of Marker-1, and then return to a stable standing position as soon as possible. Five trials were performed for each task. Rests were taken between each round of data collection for as long as requested by the participant (Hondzinski et al., 2010). Before data collection, the participants had 10 min to familiarize the test protocol, and their height, weight, dominant arm length, and shoulder height were recorded. The protocol was similar to a previous study, in which the effects of aging and sensory deficits were examined (Hondzinski et al., 2010).

#### Proprioception test

The proprioception threshold at the ankle joint of the dominant leg was assessed using a proprioception test device (AP-II, Sunny Co. Ltd., Jinan, China) (Figure 1C). Good test-retest reliability (ICC value, 0.74∼0.94) for the device has been reported previously (Sun et al., 2015). The dominant leg was defined as the preferred leg for kicking a football in the lab. The minimum angular motion that the patient can detect during ankle dorsiflexion/plantarflexion was collected by using the proprioception test device. The device consists of a box and a platform that can rotate within the frontal and sagittal planes. Two electric motors drive the platform at an angular velocity of 0.4°/s (Song et al., 2021). The movement of the platform can be stopped at any time by a hand switch controlled by the participants. An electronic goniometer in the device recorded the angular displacement of the platform. Each participant was seated on a height-adjustable chair with the foot placed on the platform. During the ankle proprioception test, the knee and hip joints were flexed at 90°, and the leg was perpendicular to the surface of the platform when the platform was placed in a horizontal position. Approximately 50% of the participant’s lower extremity weight was rested on the platform using the thigh cuff suspension system to control unwanted sensory cues from the contact between the platform and the plantar surface of the foot. The participant sat with their eyes closed and wore headphones with light music playing to eliminate potential environmental visual and auditory stimulation. The participant was instructed to concentrate on their foot and press the hand switch to stop the movement of the platform when they could sense motion, followed by identification of the rotation direction. The motor was operated to rotate with a random time interval ranging from 2 to 10 s after an indication to start a trial. At least five trials were performed for each direction to reduce random measurement errors.

#### Tactile sensation test

The dominant foot’s tactile sensation was tested with the participants while lying supine on the treatment table with a set of Semmes-Weinstein monofilaments (six piece foot kit, North Coast Medical, Inc., Morgan Hill, CA, United States) (Figure 1D), which showed good test-retest reliability (ICC value, 0.83∼0.86) (Collins et al., 2010). Monofilaments of 6 different sizes used in this study were 2.83, 3.61, 4.31, 4.56, 5.07, and 6.65 that applies 0.07, 0.4, 2, 4, 10, and 300 grams of force when being pressed into a C-shape (bent 90°). The filament size was log10 (10 × force in milligrams). The filaments were applied to the skin on the bases of the great toe, 1st and 5th metatarsals, arch, and heel in random order (Song et al., 2021). These touches were performed for 1 s and with two repetitions. Randomized null-stimuli were added to ensure that the participants could not anticipate the application of the filaments. Plantar sensitivity was determined by the initial application of the thin filaments, progressing to the thicker filaments until the participants were able to detect the touch (Song et al., 2021). The participants were asked to provide a verbal response about the localization of the area tested when they perceived the stimulation. The sensitivity threshold was determined by the minimum monofilament gauge detected correctly. A less sensitivity threshold indicates better plantar tactile sensation.

### Data reduction

Force plate data were used in calculating ground reaction force (GRF) and D_max_ of COP trajectory. COP was measured in the anterior-posterior (AP) and mediolateral (ML) directions. The GRF and COP data were filtered using a lowpass fourth-order Butterworth digital filter with a cut-off frequency of 50 Hz (Pan et al., 2016). The 20 s GRF data after the Marker-2 detached from the Marker-1 (or detached from the position of the removed Marker-1) were used in calculating the TTS. Two time-windows of the last 10 s (10∼15 s, 15∼20 s) of the AP and ML components of the GRF were analyzed. The windows with the smallest absolute GRF range for the AP and ML components were regarded as the optimal range of variation values (Ross and Guskiewicz, 2004). The 20 s COP data were collected from each participant after they began to move Marker-2 to Marker-1 (or removed Marker-1) (Prieto et al., 1996; Pan et al., 2016). The hand movement onset was taken as the moment when hand velocity exceeds 5% of the maximal hand speed at the beginning of the movement (Kubicki et al., 2012). The hand movement offset was calculated when the hand velocity fell below 5% of the maximal hand speed (the maximum speed of the Marker M2) at the end of the movement (Kubicki et al., 2012). Marker-2 position data were filtered with a 5 Hz fourth-order lowpass Butterworth filter (Hondzinski et al., 2010) and used in calculating TPE in the AP, ML, and vertical directions and three-dimensional space.

### Variables

The TPE was calculated in the EXCEL as the distance between a participant’s pointed position by memory (Marker-2) and the position of Marker-1 before it was removed. The TPE in 3D space is the 3D spatial distance between the marker 2 and the position of Marker-1 before it was removed. Final Marker-2 location was determined as the average of five frames after the movement offset (Hondzinski et al., 2010). TTS was defined as the time from Marker-2 detached from Marker-1 until the body regains stability, i.e., the starting moment when the smoothed GRF was within the optimal range of variation values for at least 0.5 s (Tulloch et al., 2012). The D_maxAP_/D_maxML_ was defined as the maximum displacement (maximum-minimum) of COP trajectory in the AP/ML direction.

### Data analysis

All statistical analysis was conducted using the SPSS software package (26.0, SPSS Inc., Chicago, IL, United States). Descriptive analysis results were presented as mean ± standard error in TTS, proprioception, and tactile sensation. The normality of all variables was tested using the Shapiro-Wilk test. Independent sample *t-*test (normally) or the Mann–Whitney U (non-normally) test was used to analyze TPE, proprioception, and tactile sensation thresholds. Two-way analysis of variance with repeated measures (normally) or Scheirer-Ray-Hare test (non-normally) was used to determine differences in TTS and D_max_ of COP trajectory.

A Bonferroni-adjusted *post-hoc* analysis was conducted when significant Group-by-Task interaction was detected. Partial eta squared (η^2^_p_) was used to represent the effect size of the main effect and interaction of the two-way analysis of variance. The thresholds for η^2^_p_ were as follows: <0.06, small; 0.06∼0.14, moderate; >0.14, large (Pierce et al., 2004). Cohen’s *d* was used to represent the effect size of the *post-hoc* pair comparison. The thresholds for Cohen’ *d* were as follows: <0.20, trivial; 0.20∼0.50, small; 0.51∼0.80, medium; >0.80, large (Cohen et al., 1988). Pearson (normally) or Spearman (non-normally) correlations were used for testing the relationships of visuospatial ability and postural stability to proprioception and tactile sensation. The thresholds for the correlation coefficient (r) were as follows: <0.10, trivial; 0.10∼0.30, weak; 0.31∼0.50, moderate; >0.50, strong (Cohen, 1988). A Type I error rate of less than 0.05 was used as an indication of statistical significance.

## Results

Of the 37 participants, 18 were included in the Tai Chi group (female = 4, male = 14, age = 69.7 ± 3.9 years, weight = 70.6 ± 11.0 kg, height = 1.68 ± 0.08 m, BMI = 24.8 ± 2.9 kg/m^2^, MMSE scores = 28.83 ± 1.01, Tai Chi experience = 10.6 ± 5.6 years), and 19 were included in the control group (female = 5, male = 14, age = 68.2 ± 3.3 years, weight = 71.4 ± 12.4 kg, height = 1.65 ± 0.08 m, BMI = 26.2 ± 3.1 kg/m^2^, MMSE scores = 27.11 ± 2.38). The basic information of each participant in Tai Chi or control groups is shown in Table 1. Independent *t*-tests showed no significant differences in age, weight, height, and BMI between the groups.

The Shapiro-Wilk test showed that most variables were normally distributed, except the TPE in the ML and vertical directions and tactile sensation thresholds.

**FIGURE 2:**
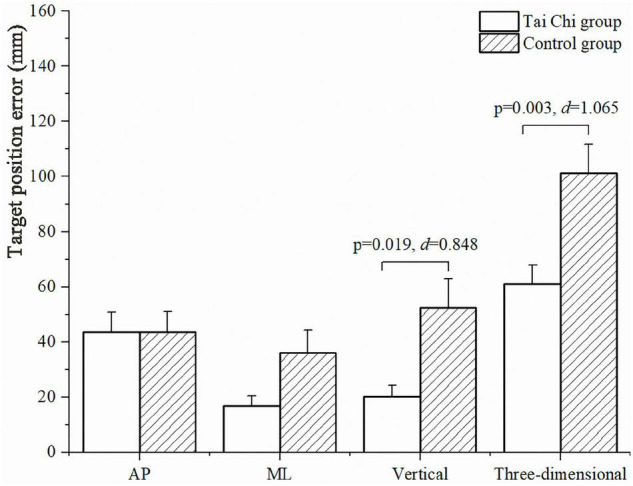
Target positioning error (TPE) between the finger marker and the removed bar marker in the AP, ML, vertical directions, and in three-dimensional space during the blind touch task (BTT). AP, anterior-posterior; ML, mediolateral.

The TPE between Marker-1 and Marker-2 is shown in Figure 2. The bar chart with error lines represented the mean and standard error of the TPE among the Tai Chi and control groups. Compared with the control group, the Tai Chi group had significantly less TPE in the vertical direction (TPE_V_; Tai Chi group: 20.3 ± 3.9 mm; control group: 34.9 ± 4.4 mm, *p* = 0.019, *d* = 0.848) and in three-dimensional space (Tai Chi group: 61.0 ± 6.9 mm; control group: 101.3 ± 10.3 mm, *p* = 0.003, *d* = 1.065) during the BTT.

The descriptive statistics and subgroup comparisons of the TTS are presented in Table 2. No Group-by-Task interaction was observed. Significant group effects were detected in TTS in the AP (TTS_AP_; *p* = 0.001, η^2^_p_ = 0.458) and ML (TTS_ML_; *p* < 0.001, η^2^_p_ = 0.557) directions. The control group took a longer time to be stabilized compared to the Tai Chi group.

**TABLE 2:** Time to stabilization (TTS) in the anterior-posterior (AP) and mediolateral (ML) directions during the touch task (TT) and blind touch task (BTT).

Variables	Task	Group		Group-by-task interaction		Task effect		Group effect	
					
		Tai Chi (*N* = 18)	Control (*N* = 19)	*p*	η^2^_p_	*p*	η^2^_p_	*p*	η^2^_p_
TTS_AP_ (s)	TT	2.63 ± 0.83	3.03 ± 0.47	0.258	0.075	0.152	0.117	**0.001**	0.458
	BTT	2.25 ± 0.66	2.93 ± 0.66						
TTS_ML_ (s)	TT	2.07 ± 0.83	3.20 ± 0.65	0.266	0.072	0.417	0.039	**<0.001**	0.557
	BTT	2.16 ± 0.75	2.88 ± 0.71						

Values are presented as mean ± SD.

Bold and highlighted: p < 0.05, compared with control group.

AP, anterior-posterior; BTT, blind touch task; ML, mediolateral; TTS, time to stabilization; TT, touch task.

**FIGURE 3:**
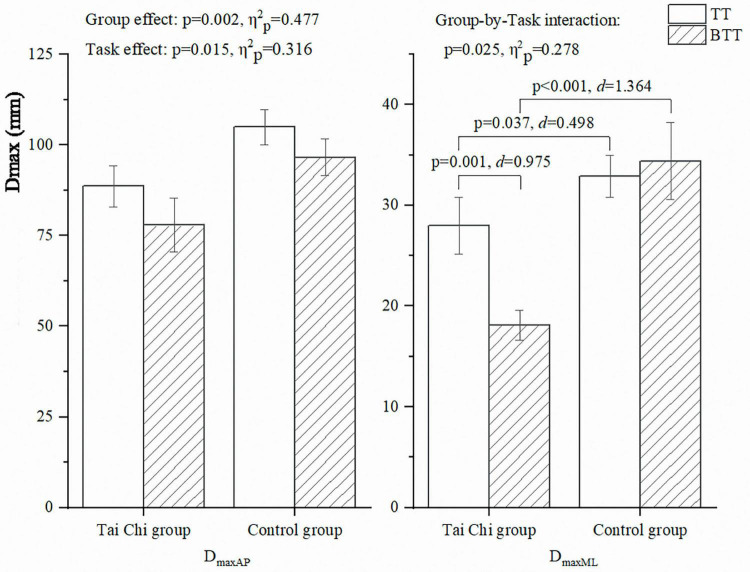
D_max_ in the AP and ML directions during the TT and BTT. AP, anterior-posterior; BTT, blind touch task; D_max_, maximum displacement; ML, mediolateral; TT, touch task.

The descriptive statistics and subgroup comparisons of D_max_ of COP trajectory in the AP (D_maxAP_) and ML (D_maxML_) directions were presented in Figure 3. Significant Group-by-Task interactions were detected in D_maxML_ (*p* = 0.025, η^2^_p_ = 0.278). Pairwise comparisons showed that D_maxML_ direction was larger during the TT than during the BTT among the Tai Chi group (*p* = 0.001, *d* = 0.975); And less during the TT (*p* = 0.037, *d* = 0.498) and BTT (*p* < 0.001, *d* = 1.364) among the Tai Chi group compared with the control group. Significant group and task effects were detected in D_maxAP_ (*p* = 0.002, η^2^_p_ = 0.477; and *p* = 0.015, η^2^_p_ = 0.316), which was larger among the control group and TT than the Tai Chi group and BTT.

The differences in ankle proprioception and tactile sensation thresholds between the TC and the control groups are presented in Table 3. Participants in the Tai Chi group had less ankle proprioception thresholds during both dorsiflexion (*p* = 0.029, *d* = 0.886) and plantarflexion (*p* = 0.012, *d* = 1.025), and less tactile sensation thresholds at the great toe (*p* = 0.015, *d* = 0.932), arch (*p* = 0.019, *d* = 0.855) and heel (*p* = 0.027, *d* = 0.930), compared to people in the control group.

**TABLE 3:** Proprioception and tactile sensation thresholds.

Sensations		Tai Chi group	Control group	*p*	*d*
Proprioception (°)	Dorsiflexion	1.58 ± 0.82	2.98 ± 2.08	**0.029**	0.886
	Plantarflexion	1.47 ± 0.73	3.24 ± 2.33	**0.012**	1.025
Tactile sensation (gauge)	Great Toe	3.93 ± 0.51	4.32 ± 0.30	0.015*	0.932
	Metatarsal 1	4.13 ± 1.04	4.38 ± 0.22	0.162	0.333
	Metatarsal 5	4.27 ± 0.25	4.48 ± 0.60	0.380	0.457
	Arch	4.10 ± 0.44	4.54 ± 0.58	0.019*	0.855
	Heel	4.25 ± 0.47	4.86 ± 0.80	0.027*	0.930

Values are presented as mean ± SD.

Bold and highlighted: p < 0.05, compared with control group.

*Significant difference from the Mann–Whitney U test, or else from the independent sample t-test.

The relationships between TPE, TTS, D_max_ and proprioception/tactile sensation thresholds are presented in Table 4. Among the Tai Chi group, sensitive ankle proprioception during plantarflexion was strongly correlated to less TPE_V_ (*r* = –0.515, *p* = 0.029), TTS_ML_ (*r* = 0.587, *p* = 0.01) and D_maxAP_ during the TT (*r* = –0.629, *p* = 0.005). Sensitive tactile sensation at the great toe was strongly correlated to less TTS_AP_ during the TT (*r* = 0.550, *p* = 0.018) and BTT (*r* = 0.564, *p* = 0.015), and moderately correlated to D_maxAP_ during the TT (*r* = –0.468, *p* = 0.05); Among the control group, sensitive ankle proprioceptions during dorsiflexion (*r* = 0.492, *p* = 0.033) and plantarflexion (*r* = 0.519, *p* = 0.021) were moderately to strongly correlated to less TTS_ml_ during the BTT.

**TABLE 4:** Relationship of TPE, TTS, D_max_ to proprioception and tactile sensation.

Correlation coefficient (r)		TPE (mm)	TTS (s)				D_max_ (mm)
			TT		BTT		TT
		Vertical	AP	ML	AP	ML	AP
**Tai Chi group**							
Proprioception (°)	Dorsiflexion	0.008	0.053	0.049	0.082	–0.383	–0.200
	Plantarflexion	**–0.515**	–0.022	**0.587**	0.313	0.003	**–0.629**
Tactile sensation (gauge)	Great toe	–0.323	0.550*	–0.003	0.564*	0.064	–0.468*
**Control group**							
Proprioception (°)	Dorsiflexion	–0.283	–0.116	0.085	0.206	**0.492**	–0.095
	Plantarflexion	–0.242	–0.050	0.038	0.182	**0.519**	0.086

AP, anterior-posterior; BTT, blind touch task; D_max_, maximum displacement; ML, mediolateral; TPE, target positioning error; TT, touch task.

Bold and highlighted: p < 0.05, significantly correlated.

*Significant correlated from Spearman correlation, or else from Pearson correlation.

Adjusted for age, weight, and height.

## Discussion

This study investigated the differences in visuospatial ability and postural stability between the Tai Chi practitioners with more than 5 years’ experience and their controls during the TT and BTT. The relationship between visuospatial ability and postural stability with proprioception and the tactile sensation was also explored. The results supported our hypotheses. The TPE was significantly less, the time to recover from an unstable state was significantly shorter, and the D_max_ was significantly less among the Tai Chi group compared to the control group. The Tai Chi group decreased D_maxML_ during the BTT compared to TT; Compared to the control group, the Tai Chi group was more sensitive in proprioception and tactile sensation.

The TPE provided the following key data: 1. The Tai Chi and control groups did not reach Marker-2 far enough to the original position of Marker-1 during the BTT; 2. Less TPE in the vertical direction and three-dimensional space were detected during the BTT among the Tai Chi group, compared to the control group; 3. The TPE was correlated to ankle plantarflexion proprioception among the Tai Chi group. The further the practitioner stretched, the more forward the center of gravity was, and the greater disturbance of postural stability was. Therefore, those practitioners selected a more conservative strategy to maintain their postural stability during the BTT and prevent falls. TPE was correlated to participants’ gaze direction (Kennedy and Inglis, 2002; Meyer et al., 2004) and sensation (Liu et al., 2012). The effects of Tai Chi practice on gaze stability (McGibbon et al., 2004), proprioception, and tactile sensation (Hu et al., 2021) had been proven, and this study further indicated that the proprioception among the Tai Chi group was correlated to the TPE. Among the Tai Chi group, the TPE was correlated to ankle proprioception during plantarflexion, but not dorsiflexion. During the tasks, the participants needed to lean forward to touch the marker, and ankle plantarflexors, such as soleus and gastrocnemius, were stretched. The proprioceptive receptors in these muscles provide spatial and temporal afferent information to maintain postural stability (Hogervorst and Brand, 1998). In the plantarflexion proprioceptive test, the same muscles were stretched and the sensitivity of proprioception was also determined by the function of the proprioceptive receptors in these muscles (Hogervorst and Brand, 1998). It is reasonable to indicate that Tai Chi practitioners used their better proprioception to reduce their TPE. The less TPE reflected the higher positioning accuracy of the Tai Chi group.

The results of TTS showed that 1. The Tai Chi group had shorter TTS than the control group; 2. TTS was correlated to proprioception and tactile sensation among the Tai Chi group and correlated to proprioception only among the control group. 3. TTS was correlated with proprioception during the BTT among the control group. TTS was defined as the time that a participant returns to a stable state from an unstable condition (Ross and Guskiewicz, 2003, 2004; Fransz et al., 2015) and could be used in evaluating postural stability (Ross and Guskiewicz, 2003). A shorter TTS indicated improved posture stability and low fall risk (Fransz et al., 2015; Sherrington et al., 2019). Individuals with functional ankle instability had longer TTS than the healthy controllers, and long TTS might be caused by poor neuromuscular functions (Ross and Guskiewicz, 2004). Our study pointed out that both proprioception and tactile sensation were correlated to TTS. Ross and Guskiewicz (2004) supported the relationship by showing that ankle joint proprioception was highly correlated to an individual’s TTS. Konradsen and Ravn (1991) suggested that proprioceptive defects at the ankle joint weaken the reflex contraction of the muscles that stabilize the body. This is the first study that revealed the relationship between tactile sensation and TTS to the best of our knowledge. Song et al. (2021) indicated that tactile sensation was only correlated to postural control when body movements were restricted. During the TT and BTT, older adults fixed their feet on the ground, so their body movements were relatively limited. In this circumstance, the position of the plantar COP changes as the subtle variation of GRFs, the perception of forces under the feet during the stance could be used to generate an internal estimate of the body center of mass location (Meyer et al., 2004). Tactile sensation afferents could provide valuable feedback to the central nervous system regarding ankle torque production, weight transfer, and limb loading (Meyer et al., 2004). From this viewpoint, a better tactile sensation might help to reduce the TTS among the Tai Chi group. In addition, this study showed that only the tactile sensation at the great toe was correlated to TTS. The tactile sensation is different between foot sole sites due to the notable differences in cutaneous receptor distribution, firing characteristics (Kennedy and Inglis, 2002), and the mechanical properties of the skin, like its hardness and thickness (Strzalkowski et al., 2015). Previous studies showed that the arch and great toe were thin and soft plantar regions (Strzalkowski et al., 2015) with better sensitivity, which was correlated to static balance control (Song et al., 2021). In our study, only tactile sensation at the great toe, rather than at the arch, was correlated with the postural stability. As one of the areas with the highest pressure during walking, great toe is subjected to much greater pressure loads than the arch (Mao et al., 2006b). It could be inferred that more pressure signals at the great toe could be transmitted to the central nervous system, so the correlation of TTS to tactile sensation at the great toe was detected, rather than at the arch. In our study, only the tactile sensation at the great toe, but not the arch, was correlated with postural stability. As one of the areas with high pressure during walking, the great toe is subjected to a much higher weight load than the arch (Mao et al., 2006b). It can be inferred that more pressure signals at the great toe could be transmitted to the central nervous system, so that significantly correlation of TTS with tactile sensation at the big toe was detected, but not at the arch. Among the control group, TTS was correlated with proprioception during the BTT, but not TT. Proprioception and vision can be compensated for each other by sensory weighting in the central nervous system (Rand et al., 2013), so the participants may rely more on proprioception to maintain postural stability during the BTT, where there was less visual information.

The outcomes of the D_max_ of COP trajectory showed that: 1. The Tai Chi group showed significantly less D_max_ in the AP direction during the TT and BTT compared with the control group. 2. The Tai Chi and control groups showed significantly less D_max_ in the AP direction during the BTT than during the TT. 3. Significant Group-by-Task interaction showed that the Tai Chi group had decreased D_max_ in the ML direction during BTT than during TT, while the control group did not have similar changes. 4. D_max_ was correlated to proprioception and tactile sensation among the Tai Chi group. One of the previous studies supported our first discovery by indicating that the Tai Chi group showed significantly less postural sway than the brisk walking and control groups during goal-directed upper body movements (Pan et al., 2016). The better postural control could explain the less D_max_ (Hondzinski et al., 2010; Zhang et al., 2011; Hu et al., 2021; Song et al., 2021). The less Dmax in the AP direction during the BTT may be explained by the fact that the Tai Chi and control groups reached their hands less forward and produced a small postural sway in the AP direction during the BTT. The Group-by-Task interaction in the D_max_ indicated that the Tai Chi group increased their postural stability in the ML direction during BTT compared to that during TT. Compared with normal walking, Tai Chi has more variety movements and greater plantar loading in the ML direction (Mao et al., 2006a), so it is not surprising that the Tai Chi group showed better postural stability in the ML direction. Compared to TT, the effects of vision on postural control were reduced during the BTT because the target was removed, and individuals relied more on sensations to compensate for the decreased vision effects. The outcomes of this and previous studies well-documented that proprioception and tactile sensation were better among the Tai Chi group (Li et al., 2019; Hu et al., 2021). It was reasonable to assume that the Tai Chi group took advantage of their better sensations to control their movements more precisely and decreased the disturbance in the ML direction during BTT than during TT.

The differences in proprioception and tactile sensation between the Tai Chi and control groups and the relationships of proprioception and tactile sensation to visuospatial ability and postural stability showed that: 1. Most of the proprioception and tactile sensation thresholds were significantly less among the Tai Chi group than among the control group; 2. Compared with the control group, more correlations of proprioception to visuospatial ability and postural stability were detected among the Tai Chi group; and 3. No correlations of tactile sensation to visuospatial ability and postural stability were detected among the control group. The positive effects of Tai Chi exercise on proprioception and tactile sensation had been extensively investigated and well-explained previously (Hondzinski et al., 2010; Zhang et al., 2011; Hu et al., 2021). We investigated the relationship of proprioception and tactile sensation to visuospatial ability and postural stability. The proprioceptive receptors in and around joints provided important spatial and temporal afferent information regarding the positions and movements of body segments and information between body segments in space (Hogervorst and Brand, 1998; Relph and Herrington, 2016). Proprioceptive receptors afferent information was transmitted to the central nervous system, and these receptors, in turn, were organized and managed in various high-order areas. Therefore, proprioception had an important role in visuospatial ability and postural stability. One study measured the postural sway in 74 healthy subjects from different age groups. It reported that all groups were more dependent on proprioception than on other sensations to maintain balance control (Colledge et al., 1994). It is reasonable to assume that individuals prefer proprioception over vision to precisely control their movements. Hence, the Tai Chi group relied more on their better proprioception and performed better during upper body movements. Moreover, our outcomes indicated that proprioception was only correlated to postural stability during BTT, but not during TT.

In this study, the tactile sensation was better among the Tai Chi group. One previous study supported our observations and further indicated that Tai Chi intervention significantly improved tactile sensation among older adults by altering ring the plasticity of the sensory-motor system to increase somatosensory information from the plantar sensory (Hondzinski et al., 2010). Skin receptors in the foot sole are sensitive to contact pressure and potential changes in the distribution of pressure, provide important information about the body’s status with respect to the supporting surface (Kennedy and Inglis, 2002). Tai Chi practitioners used their better tactile sensation, felt the slight changes of plantar pressure distribution, and finally improved their visuospatial ability and postural stability. This study confirmed the relationship of visuospatial ability and postural stability to proprioception and tactile sensation. This finding has clinical implication. Exercises that enhance sensations should be included in the exercise prescriptions for fall prevention among older adults.

This study has several limitations. First, considering the size of the sample and the heterogeneity in the number of years of practicing Tai Chi, it could be specified that it is an exploratory study. Second, this is an analytical cross-sectional study, rather than a randomized controlled trial, only one measurement of the study variables was made, and no variables were assessed over time, that might cause some participant selection bias. Therefore, future studies should be designed by randomized controlled trials. Third, this study only examined the relationship of proprioception and tactile sensation with the visuospatial ability and postural stability, other contributors, such as the central nervous system, visual, vestibular, or cognitive functions, could also influence them. Fourth, some observations of this study could not to be explained by our limited understanding, e.g., only proprioception during plantarflexion, instead of during dorsiflexion, was correlated to visuospatial ability and postural stability among the Tai Chi group, and only proprioception during the BTT, instead of during the TT, was correlated to postural stability among the control group. Therefore, further investigations can be designed based on our new and innovative report.

## Conclusion

During goal-directed upper body movements, long-term Tai Chi practitioners exhibited superior visuospatial ability and superior postural stability, along with better proprioception and tactile sensation. Compared with those of the control group, Tai Chi practitioners’ better sensations enhanced their superior visuospatial ability and postural stability.

## Data availability statement

The raw data supporting the conclusions of this article will be made available by the authors, without undue reservation.

## Ethics statement

The studies involving human participants were reviewed and approved by Sports Science Ethics Committee of Shandong Sport University. The patients/participants provided their written informed consent to participate in this study. Written informed consent was obtained from the individual(s) for the publication of any identifiable images or data included in this article.

## Author contributions

ZS participated in the design of the study and contributed to the data collection, reduction, and analysis. LL and QS participated in the design of the study. WS participated in the design of the study and contributed to the data collection. MM contributed to the data reduction and analysis. CZ contributed to the data analysis and interpretation of results. All authors contributed to the manuscript writing, read and approved the final version of the manuscript, and agreed with the order of presentation of the authors.

## Conflict of interest

The authors declare that the research was conducted in the absence of any commercial or financial relationships that could be construed as a potential conflict of interest.

## Publisher’s note

All claims expressed in this article are solely those of the authors and do not necessarily represent those of their affiliated organizations, or those of the publisher, the editors and the reviewers. Any product that may be evaluated in this article, or claim that may be made by its manufacturer, is not guaranteed or endorsed by the publisher.
